# Effects of blood lead on coronary artery disease and its risk factors: a Mendelian Randomization study

**DOI:** 10.1038/s41598-019-52482-1

**Published:** 2019-11-05

**Authors:** C. Mary Schooling, Glen D. Johnson, Jean Grassman

**Affiliations:** 1 Graduate School of Public Health and Health Policy, City University of New York, New York, United States; 2 School of Public Health, Li Ka Shing Faculty of Medicine, The University of Hong Kong, Hong Kong Special Administrative Region, Hong Kong, China

**Keywords:** Cardiology, Cardiovascular biology, Risk factors

## Abstract

Lead is pervasive, although lead exposure has fallen in response to public health efforts. Observationally, lead is positively associated with cardiovascular disease and hypertension. We used separate-sample instrumental variable analysis with genetic instruments (Mendelian randomization) based on 13 single nucleotide polymorphisms (SNP), from a genome wide association study, strongly (p-value < 5 × 10^−6^) and independently associated with blood lead. These SNPs were applied to a large extensively genotyped coronary artery disease (CAD) study (cases = <76014, controls = <264785) largely based on CARDIoGRAPMplusC4D 1000 Genomes and the UK Biobank SOFT CAD, to the UK Biobank (n = 361,194) for blood pressure and to the DIAGRAM 1000 genomes diabetes case (n = 26,676)-control (n = 132,532) study. SNP-specific Wald estimates were combined using inverse variance weighting, MR-Egger and MR-PRESSO. Genetically instrumented blood lead was not associated with CAD (odds ratio (OR) 1.01 per effect size of log transformed blood lead, 95% confidence interval (CI) 0.97, 1.05), blood pressure (systolic −0.18 mmHg, 95% CI −0.44 to 0.08 and diastolic −0.03 mmHg, 95% CI −0.09 to 0.15) or diabetes (OR 0.98, 95% CI 0.92 to 1.03) using MR-PRESSO estimates corrected for an outlier SNP (rs550057) from the highly pleiotropic gene *ABO*. Exogenous lead may have different effects from endogenous lead; nevertheless, this study raises questions about the role of blood lead in CAD.

## Introduction

Lead is a widespread environmental contaminant well-known to adversely affect cognitive development. Concerted public health action to reduce environmental exposure to lead has substantially reduced blood lead levels in developed countries^1,2^. Nevertheless, low-level lead exposure remains a risk factor for cardiovascular disease^3,4^. A direct and linear relation of lead with blood pressure has been observed^5^, possibly operating via renin and aldersterone^6^. However, whether lead is a causal target of intervention or a biomarker of other exposures that cause cardiovascular disease is unclear. Observational studies are open to confounding by a myriad of factors, including socio-economic position, lifestyle and ill-health, particularly as poorer people often more vulnerable to cardiovascular disease may have no option but to live in more polluted environments. A randomized trial of chelation showed cardiovascular benefits^7^, but chelation reduces many metals^7^, so it is difficult to know whether the benefits are due to lead reduction or other factors. As such, whether lead is a potential target of intervention for the leading cause of global morbidity and mortality remains unclear.

In these circumstances, where definitive experimental evidence is not available, but public health is at stake, one way forward is to compare the risk of disease in people with genetically different lead levels thereby taking advantage of genetic randomization at conception. As such, Mendelian randomization (MR), instrumental variable analysis (IVA) with genetic instruments, provides a means of obtaining unbiased estimates from observational studies^8^, when all assumptions are met. To our knowledge, the role of lead in coronary artery disease (CAD) has not been assessed using MR. To address this gap we assessed the role of lead in CAD, blood pressure and diabetes, using the largest available, suitable genetic studies.

## Results

### Genetic associations with blood lead

Thirteen uncorrelated SNPs rs12136530 (*CAPZB*), rs2662776 (*RGS5*), rs76153987 (*SRGAP3*), rs9863067 (*GBE1-CADM2*), rs79019069 (*AGTR1/CPB1*), rs116864947 (*THSD7A*), rs6462018 (*EVX1-H1BADH*), rs798338 (*MAGI2*), rs60580184 (*TTC26*), rs1805313 (*ALAD*), rs550057 (*ABO*), rs144653651 (*PTPN2-SEH1L*) and rs16968074 (*PEPD*)) strongly associated with blood lead (effect sizes of log transformed values) were obtained from a GWAS. All these SNPs had F-statistics > 20. One SNP (rs9863067) was palindromic, and was replaced by rs7625182 (r^2^ = 1) for diabetes, because effect allele frequency is not given for this outcome. When we checked for associations with potential confounders in the UK Biobank, one SNP rs16968074 (*PEPD*) was associated with alcohol use frequency at Bonferroni corrected significance, but the other SNPs were not found associated with potential confounders. One SNP (rs550057) from the *ABO* gene was listed in curated genotype to phenotype cross-references (Ensembl and PhenoScanner) as strongly linked with CAD and several of its risk factors. The role of *ABO* gene products in cardiovascular disease is not fully understood. We repeated the analysis excluding rs16968074 and rs550057. Given the SNPs explained ~6% of the variance of blood lead, estimates of the order of an odds ratio of 1.05 for CAD, an odds ratio of 1.08 for diabetes, a difference of 0.2 mm Hg in diastolic blood pressure and a difference of 0.4 mm Hg in systolic blood pressure per effect size of log-transformed blood lead could be detected at 80% power with 5% alpha.

### Association of blood lead with CAD, blood pressure and diabetes

**Figure 1:**
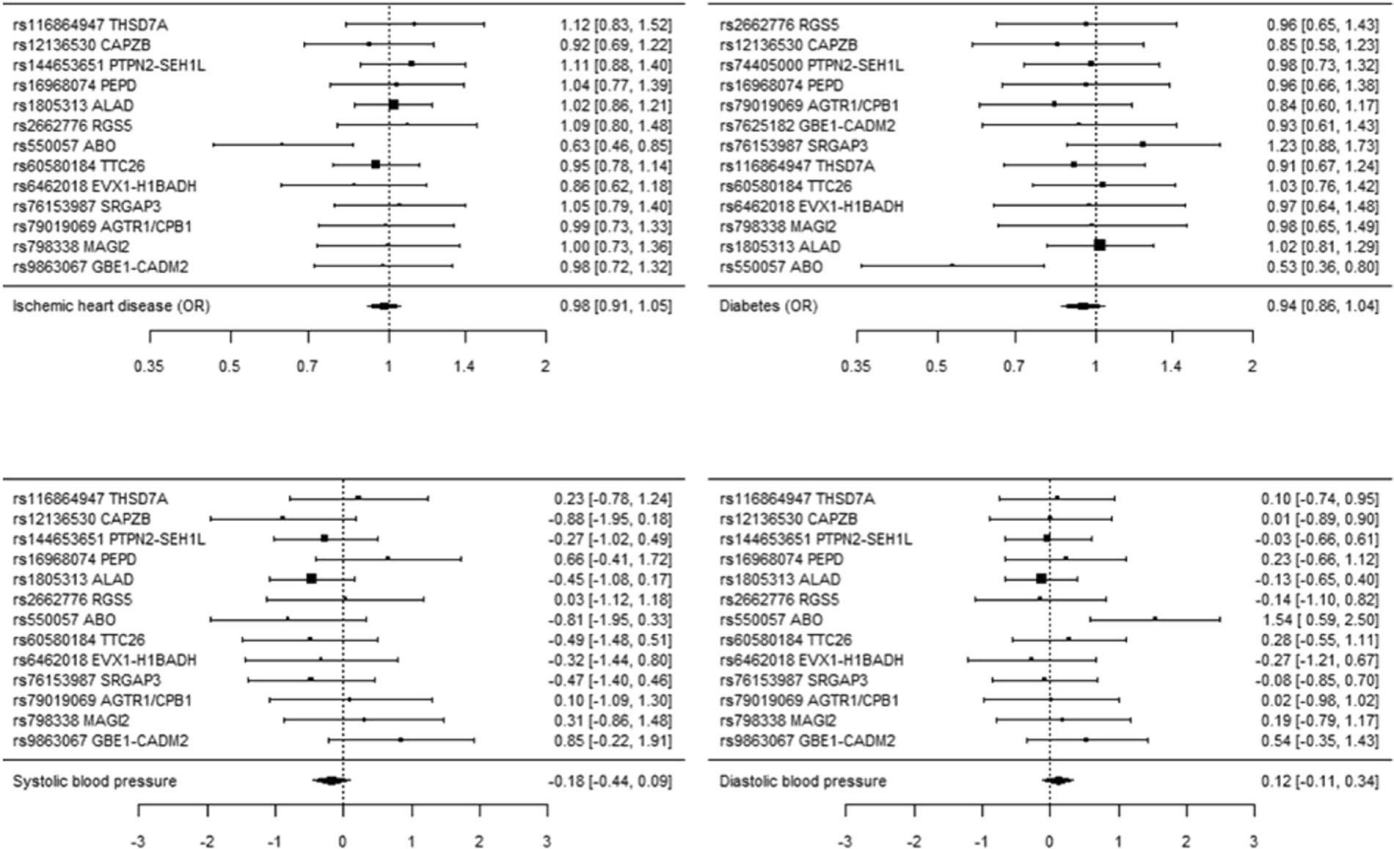
SNP-specific and overall Mendelian randomization associations of blood lead (effect sizes of log transformed blood lead) (based on 13 independent SNPs, p-value < 5 × 10^−6^) with CAD, using a study largely based on CARDIoGRAPMplusC4D 1000 Genomes and the UK Biobank SOFT CAD, with diabetes using DIAGRAM, and with blood pressure (mmHg) using the UK Biobank.

Table 1 shows genetically instrumented blood lead was generally unrelated to CAD, blood pressure and diabetes using IVW or WM. However, the Cochran’s Q statistic indicated high heterogeneity, with rs550057 visually obvious as an outlier on the forest plots for CAD, diabetes and diastolic blood pressure (Fig. 1). The MR-Egger intercepts were not different from the null value. The MR-Egger estimates were similar to those from IVW and WM, albeit with wider confidence intervals (Table 1). MR-PRESSO identified rs550057 as an outlier. The corrected estimates from MR-PRESSO (Table 1) were quite similar to the more homogenously null results obtained from the other methods after excluding rs550057 and rs16968074 (Table 2).

**Table 1:** Mendelian randomization associations of blood lead (effect sizes of log transformed blood lead) based on 13 independent SNPs (p-value < 5 × 10^−6^) with CAD, using a case-control study largely based on CARDIoGRAPMplusC4D 1000 Genomes and the UK Biobank SOFT CAD, with diabetes using Diagram, and with blood pressure using the UK Biobank.

Outcome	Mendelian Randomization Method	Odds ratio	95% confidence interval	p-value	Cochran’s Q statistic (p-value)	MR-Egger	
						Intercept p-value	I^2^
CAD	Inverse variance weighted	0.98	0.91 to 1.05	0.61	35.8 (<0.001)		
	Weighted median	1.01	0.95 to 1.07	0.85			
	MR-Egger	1.08	0.92 to 1.26	0.36	31.2 (0.001)	0.20	15%
	MR-PRESSO (corrected)	1.01	0.97 to 1.05				
Diabetes	Inverse variance weighted	0.94	0.86 to 1.04	0.23	27.4 (0.007)		
	Weighted median	0.97	0.89 to 1.06	0.54			
	MR-Egger	1.04	0.83 to 1.28	0.75	25.3 (0.008)	0.35	0%
	MR-PRESSO (corrected)	0.98	0.92 to 1.03				
		β					
SBP	Inverse variance weighted	−0.18	−0.45 to 0.09	0.20	18.5 (0.10)		
mmHg	Weighted median	−0.31	−0.62 to −0.003	0.048			
	MR-Egger	−0.27	−0.94 to 0.39	0.42	18.4 (0.07)	0.75	4.8%
	MR-PRESSO	−0.18	−0.44 to 0.08				
DBP	Inverse variance weighted	0.12	−0.11 to 0.34	0.31	39.8 (<0.0001)		
mmHg	Weighted median	−0.01	−0.18 to 0.17	0.94			
	MR-Egger	−0.04	−0.58 to 0.51	0.90	38.5 (<0.0001)	0.54	4.8%
	MR-PRESSO (corrected)	0.03	−0.09 to 0.15				

rs9863067 replaced by proxy rs7625182 for diabetes.

**Table 2:** Mendelian randomization associations of blood lead (effect sizes of log transformed blood lead) based on 11 (rs16968074 and rs550057 excluded) independent SNPs (p-value < 5 × 10^−6^) with CAD using a study largely based on CARDIoGRAPMplusC4D 1000 Genomes and the UK Biobank SOFT CAD, with diabetes using Diagram, and with blood pressure using the UK Biobank.

Outcome	Mendelian Randomization Method	Odds ratio	95% confidence interval	p-value	Cochran’s Q statistic (p-value)	MR-Egger	
						Intercept p-value	I^2^
CAD	Inverse variance weighted	1.01	0.96 to 1.05	0.82	9.9 (0.45)		
	Weighted median	1.01	0.95 to 1.07	0.87			
	MR-Egger	1.05	0.95 to 1.16	0.38	9.1 (0.43)	0.38	27%
	MR-PRESSO (corrected)	1.01	0.96 to 1.05				
Diabetes	Inverse variance weighted	0.98	0.91 to 1.04	0.50	8.6 (0.57)		
	Weighted median	0.98	0.90 to 1.07	0.68			
	MR-Egger	0.99	0.85 to 1.14	0.87	8.5 (0.48)	0.86	1%
	MR-PRESSO (corrected)	0.98	0.92 to 1.04				
		β					
SBP	Inverse variance weighted	−0.20	−0.46 to 0.07	0.14	13.1 (0.22)		
mmHg	Weighted median	−0.32	−0.64 to 0.004	0.05			
	MR-Egger	−0.26	−0.88 to 0.38	0.43	13.0 (0.16)	0.84	17.7%
	MR-PRESSO	−0.20	−0.46 to 0.07				
DBP	Inverse variance weighted	0.02	−0.11 to 0.15	0.77	8.9 (0.54)		
mmHg	Weighted median	−0.03	−0.21 to 0.14	0.75			
	MR-Egger	0.12	−0.18 to 0.42	0.32	12.7 (0.18)	0.44	18.7%
	MR-PRESSO (corrected)	0.02	−0.11 to 0.15				

rs9863067 replaced by proxy rs7625182 for diabetes.

## Discussion

Our study does not suggest that blood lead plays a major role in CAD or blood pressure. Here, we used an increasingly popular study design that generates unbiased estimates by exploiting existing resources without any exposure to humans to assess the effects of blood lead on CAD, blood pressure and diabetes. Using different samples for the exposure and the outcomes reduces the risk of confounding by a shared data structure. Using samples largely from people of European descent with appropriate genomic control^9,10^ reduces the risk of bias from population stratification. Use of very large samples means the study had power to detect very small effect sizes.

Despite these advantages, IVA has three key assumptions, which cannot all be empirically verified. The genetic predictors should be strongly associated with the exposure. We used p-value < 5 × 10^−6^ as a cut-off, and we checked the F-statistics were > 10. One of the SNPs (rs1805313) came from the *ALAD* gene known to be relevant to lead metabolism^10^, which suggest some physiological validity as an instrument, but rs1805313 was not associated with the outcomes considered (Fig. 1). In addition, the ALAD gene is not associated with CAD^11^. Nevertheless, the GWAS for blood lead was relatively small (n = 5433) with only one genome wide significant SNP which could have led to weak instrument bias towards the null in the two-sample context^12^, for example if the instruments included false positives or picked up noise instead of signal. No confounders of genetic predictors on outcome should exist, which we tested in the UK Biobank, and repeated the analysis excluding a SNP (rs16968074) possibly associated with alcohol use. The genetic predictors should only affect CAD, blood pressure and diabetes via lead. We checked for known pleiotropy using Ensembl and PhenoScanner, and found few known phenotypes of the genetic predictors of blood lead apart from multiple associations for rs550057 (*ABO*). We checked for unknown pleiotropy using MR-Egger and MR-PRESSO. MR-PRESSO also identified rs550057 as a potentially pleiotropic outlier. Finally, although less commonly stated, IVA assumes that the association of SNPs with exposure and outcome are free from selection bias. Genetic studies are open to bias from survival^13^ which is compounded if common causes of survival and outcome exist^14^. The GWAS of blood lead, CAD, blood pressure and diabetes are in relatively young people and do not share many common causes with conditions that cause death at earlier ages making such selection bias less likely. However, we cannot totally exclude the possibility that the null results are explained in part by prior death from these diseases of those vulnerable to the effects of lead.

This study largely concerns people of European descent. Lead may have different effects in different populations, although lead is not known to operate by population specific mechanisms. It is also possible that the levels of blood lead in these populations were too low to be relevant to cardiovascular disease, however associations with cardiovascular disease at very low levels have been reported^3^. Using publicly available data precludes subgroup analysis by sex, age and baseline levels of blood lead. We could not replicate using another GWAS of blood lead because the only other GWAS is unclear on effect allele^15^.

The CAD case-control study used is not composed entirely of incident cases, but includes prevalent cases enriched for early onset CAD. As such, any associations may pertain to factors driving survival rather than factors causing CAD, which could be relevant to the lack of association. Replication using incident cases would be helpful. We assumed relations of blood lead with CAD, diabetes and blood pressure are linear. Canalization might compensate for any genetic effects, however the extent of canalization is not known. Finally, lead in blood or erythrocytes does not represent the total body burden of lead that may be sequestered elsewhere, particularly in bone. However, circulating blood lead is more likely relevant to systemic diseases, such as CAD and hypertension.

Lead may act on red blood cells which also may have a role in CAD^16^. *In vivo* and *in vitro* experiments suggest lead may increase oxidative stress, inflammation, atrial natriuretic peptide and endothelin while decreasing nitric oxide^17^. Endothelin-1 and nitric oxide have genetically validated effects on CAD^9,18^. Conversely, cardiovascular disease is increasing being placed within well-established theoretical constructs, such as the evolutionary biology theory seen across the animal kingdom that longevity trades-off against growth and reproduction^19,20^, with genetic selection in favour of both CAD and fertility observed^21^. Lead is known for its testicular toxicity^22^. As such, blood lead may act via a variety of mechanisms with both detrimental and protective effects on CAD and blood pressure.

## Conclusion

We found lead unrelated to CAD, diabetes and blood pressure. More investigation is required into the role of lead in preventing the leading cause of global morbidity and mortality.

## Methods

### Blood lead

Genetic associations with blood lead were taken from a genome wide association study (GWAS) of twins and their families (n = 2603, mean age 47.2 years, 59% women) from the Queensland Institute of Medical Research (QIMR), Australia, and of 2830 unrelated mothers (mean age 28.4 years) from the Avon Longitudinal Study of Parents and Children (ALSPAC)^10^. The QIMR study estimated genetic associations with log-transformed standardized residuals of erythrocyte lead adjusted for sex and age using an additive model accounting for within-family relatedness^10^. ALSPAC estimated genetic associations with log transformed residuals of blood lead^10^. Mean blood lead was 4.01 μg/dl^10^. Erythrocyte lead can be converted into blood lead based on average hemoglobin^10^, meaning erythrocyte and blood lead residuals represent equivalent units of blood lead which can be analyzed together.

We used all single nucleotide polymorphisms (SNPs) which independently (r^2^ < 0.05) and strongly (p-value < 5 × 10^−6^) predicted blood lead. We replaced SNPs predicting blood lead but not available for the outcomes with highly correlated proxies. We ensure palindromic SNPs were aligned on allele frequency, and used proxies as needed. We checked the strength of genetic instruments from the F-statistic using an approximation^23^; an F-statistic < 10 indicates a weak instrument. We identified any of the selected SNPs that were associated with potential confounders, i.e., smoking, education, alcohol use and physical activity, at Bonferroni corrected significance in the UK Biobank summary statistics (http://www.nealelab.is/uk-biobank/). To satisfy the IVA ‘exclusion-restriction’ assumption we searched two curated genotype to phenotype databases, Ensembl release 91 (http://useast.ensembl.org/index.html) and PhenoScanner^24^, for paths by which the SNPs predicting blood lead might affect CAD, blood pressure or diabetes other than via blood lead. Ensembl gives SNP phenotypes, PhenoScanner also gives phenotypes of correlated SNPs. We repeated the analysis excluding any SNPs so identified as potentially pleiotropic.

### Outcomes

Genetic associations with CAD were obtained from the largest publicly available extensively genotyped CAD case (n = up to 76014)-control (n = up to 264785) study based on a meta-analysis of the CARDIoGRAPMplusC4D 1000 Genomes case (n = 60,801)-control (n = 123,504) study/ MIGen/CARDIoGRAM Exome chip study, the UK Biobank SOFT CAD study (cases = 10,801, controls = 137,371), and two small case (n = 4120)-control (n = 3910) studies from Germany and Greece^25^. CARDIoGRAMplusC4D 1000 Genomes participants are largely of European descent (77%) with detailed phenotyping of CAD, MI or both, based on medical records, clinical diagnosis, procedures that indicate CAD, such as revascularization, and/or angiographic evidence of stenosis, and sometimes case status ascertained from medications or symptoms that indicate angina or from self-report^9^. The UK Biobank recruited 502,713 adults intended to be aged 40–69 years from Great Britain between 2006 and 2010, 94% self-reported European ancestry. In the UK Biobank SOFT CAD GWAS the case phenotype included fatal or nonfatal MI, percutaneous transluminal coronary angioplasty or coronary artery bypass grafting, chronic CAD and angina. Controls were defined as individuals who were not a case after exclusions^25^. Genetic associations with blood pressure (mmHg) were taken from the UK Biobank summary statistics of (n = 361,194) people of British ancestry, adjusted for sex, age, age^2^, sex*age, sex*age^2^, and 20 principal components for ancestry http://www.nealelab.is/uk-biobank/. Genetic associations with diabetes were obtained from the DIAGRAM 1000 Genomes diabetes case (n = 26,676)-control (n = 132,532) study^26^, which is the largest publicly available densely genotyped GWAS, consisting mainly of people of European descent using genomic control^26^. The mean age in the DIAGRAM study was 57.4 years, and genetic associations were adjusted for age, sex, study specific covariates and principal components for ancestry^26^.

### Statistical analysis

In the primary analysis, SNP-specific Wald estimates were combined using inverse variance weighting (IVW) with multiplicative random effects, which assumes balanced pleiotropy. Corresponding forest plots were generated from fixed effects meta-analysis by multiplying each SNP-specific variance by Cochran’s Q/(k-1). A weighted median (WM) was also used, which may provide correct estimates even when the instruments, i.e., SNPs, are invalid for up to 50% of the weight. Wald estimates were calculated by dividing the estimate for SNP on outcome by the estimate for SNP on blood lead. The variance of the SNP-specific estimates was approximated by the first term of Fieller’s theorem. We aligned palindromic SNPs on allele letter and frequency of the reference and effect alleles. SNPs that could not be unequivocally aligned were replaced by suitable proxies or dropped.

### Power analysis

We calculated power using the approximation that the sample size required for an MR study is the sample size for exposure on outcome divided by the r^2^ for instrument on exposure^27^, using a utility^28^. We obtained the r^2^ using an approximation, by summing the SNP specific r^2^ calculated as 2 × effect allele frequency (EAF) × (1 − EAF) × coefficient for SNP on blood lead squared^29^.

### Sensitivity analysis

As sensitivity analysis, we used MR-Egger and Mendelian randomization pleiotropy residual sum and outlier (MR-PRESSO). MR-Egger and MR-PRESSO may provide correct estimates as long as the instrument strength independent of direct effect assumption is satisfied. MR-Egger can be imprecise, particularly if the associations for SNPs on exposure are similar^30^, or the number of genetic instruments is low. A non-null MR-Egger intercept suggests that the IVW estimate is invalid. MR-Egger does not explicitly identify outliers. MR-PRESSO detects, and if necessary, corrects for potentially pleiotropic outliers^31^.

We used the R packages “MendelianRandomization” and “MRPRESSO” to obtain the estimates. We used R (version 3. 4. 2) for all our analysis.

### Ethics

We used publicly available summary data with no direct involvement of participants in the study. No ethical approval is required.

## Data Availability

The datasets analyzed in this study are publicly available summary statistics.
